# Advanced CNC/PEG/PDMAA Semi-IPN Hydrogel for Drug Delivery Management in Wound Healing

**DOI:** 10.3390/gels8060340

**Published:** 2022-05-30

**Authors:** Samia Afrin, Md. Shahruzzaman, Papia Haque, Md. Sazedul Islam, Shafiul Hossain, Taslim Ur Rashid, Tanvir Ahmed, Makoto Takafuji, Mohammed Mizanur Rahman

**Affiliations:** 1 Department of Applied Chemistry and Chemical Engineering, Faculty of Engineering and Technology, University of Dhaka, Dhaka 1000, Bangladesh; saa292@pitt.edu (S.A.); papiahq@du.ac.bd (P.H.); sazid.acce@du.ac.bd (M.S.I.); shossain2@huskers.unl.edu (S.H.); turashid@ncsu.edu (T.U.R.); tanvirahmed@du.ac.bd (T.A.); mizanur.rahman@du.ac.bd (M.M.R.); 2 Department of Chemistry, University of Pittsburgh, Pittsburgh, PA 15260, USA; 3 Department of Chemistry and Biochemistry, Florida State University, Tallahassee, FL 32306, USA; 4 Department of Chemical Engineering and Polymer Science, Shahjalal University of Science and Technology, Sylhet 3114, Bangladesh; 5 Department of Chemistry, University of Nebraska-Lincoln, Lincoln, NE 68588, USA; 6 Fiber and Polymer Science, North Carolina State University, Campus Box 7616, Raleigh, NC 27695, USA; 7 Department of Applied Chemistry and Biochemistry, Kumamoto University, Kumamoto 860-8555, Japan; takafuji@kumamoto-u.ac.jp

**Keywords:** semi-IPN, hydrogel, drug delivery, wound healing

## Abstract

A Semi Interpenetrating Polymer Network (semi-IPN) hydrogel was prepared and loaded with an antibiotic drug, gentamicin, to investigate the wound healing activity of this system. The semi-IPN hydrogel was synthesized by combining natural polymer cellulose nanocrystal (CNC) and synthetic polymer polyethylene glycol (PEG) and poly (N,N′-dimethyl acrylamide) (PDMAA), which was initially added as a monomer dimethyl acrylamide (DMAA). CNC was prepared from locally obtained jute fibers, dispersed in a PEG-NaOH solvent system and then mixed with monomer DMAA, where polymerization was initiated by an initiator potassium persulphate (KPS) and cross-linked by N,N′-methylenebisacrylamide (NMBA). The size, morphology, biocompatibility, antimicrobial activity, thermal and swelling properties of the hydrogel were investigated by different characterization techniques. The biocompatibility of the hydrogel was confirmed by cytotoxicity analysis, which showed >95% survival of the BHK-21, Vero cell line. The drug loaded hydrogel showed antimicrobial property by forming 25 and 23 mm zone of inhibition against *Staphylococcus aureus* (gram-positive) and *Escherichia* *coli* (gram-negative) bacteria, respectively, in antimicrobial analysis. At pH 5.5, 76% of the drug was released from the hydrogel within 72 h, as observed in an in vitro drug release profile. In an in vivo test, the healing efficiency of the drug loaded hydrogel was examined on a mice model with dorsal wounds. Complete healing of the wound without any scar formation was achieved in 12 days, which revealed excellent wound healing properties of the prepared drug loaded semi-IPN hydrogel. These results showed the relevance of such a system in the rapid healing of acute wounds.

## 1. Introduction

Hydrogels are soft, wet and cross-linked three-dimensional networks of hydrophilic polymeric materials that are capable of absorbing large amounts of water or biological fluids, and thus resemble, to a large extent, a biological tissue [1]. These hydrogels have been studied for a wide range of pharmaceutical, biomedical and daily care applications, such as drug delivery, contact lenses, tissue engineering and superabsorbent agents [2]. However, they can be hard to handle, may be difficult to load with drugs and sterilize and usually have low mechanical strength [3]. To overcome this type of problem, an Interpenetrating Polymer Network (IPN) is introduced, which is a category of such newly developed bioactive materials that are significantly used in the pharmaceutical industry [4].

IPN, in general, is a combination of two polymers in a physically cross-linked network where chains of one polymer are entangled with or penetrate the network of another polymer. Among different types of IPNs, semi-IPNs are the new systems for application in drug delivery [5]. In semi-IPN, two polymers are independent of each other while being physically interlocked and, hence, the improvement in mechanical stability of the hydrogel is possible due to physical entanglement and network interactions. Hydrogels prepared from natural polymers may offer great advantages including outstanding biocompatibility, biodegradability and low toxicity. However, these polymers also show poor mechanical properties and brittleness and, hence, cannot withstand the forces imposed in vivo, which may restrict their use in different biomedical applications. On the other hand, hydrogels synthesized from synthetic polymers generally present good mechanical properties, good process ability, easily tunable molecular weight and chemical composition. Relatively long response times, however, were required by most of them for external environment change due to slow diffusion of water. In addition, they may lack informational structure for biological response. However, the fusion of natural and synthetic polymers in the form of semi-Interpenetrating polymer network (semi-IPN) hydrogels may combine the most useful characteristics of both the systems and may address the individual drawbacks [6].

Cellulose nanocrystal (CNC) attracts a wide variety of applications in drug delivery system by incorporating a suitable drug blended with any synthetic polymer. Poly (N,N′-dimethyl acrylamide) (PDMAA), a hydrophilic synthetic biocompatible polymer, finds various applications in DNA sequencing, molecular biology, medical and pharmaceutical fields, including contact lenses and drug delivery [7]. Another polymer, polyethylene glycol (PEG), can be used as a solvent system for dispersion of CNC by grafting of PEG onto the CNC surface as PEG is a steric stabilizer in the preparation of dispersible nanoparticles [8]. Thus, the fusion of these polymers forming semi-IPN can be a great development for drug delivery systems, as it will decrease the brittleness of the corresponding CNC and, with synthetic PEG and PDMAA, it forms a matrix for semi-IPN which results in better drug loading.

Wounds occur when a tissue is disrupted or the cellular integrity is compromised due to mechanical, physical or metabolism-related issues [9]. In recent years, different bacterial contaminations of skin wounds have become very common, with high rates of morbidity and mortality [10]. Therefore, an ideal antibacterial wound dressing is necessary for this purpose. In ideal wound dressing, a moist environment around the wound is maintained and the exudates from the wound surface are absorbed. As semi-IPN hydrogels absorb a high quantity of water, a moist environment can be provided to the wound area and, moreover, hydrogels can easily absorb the exudates [11]. To improve antimicrobial properties of the wound dressing, different antibiotics can be incorporated into the hydrogel. As an antimicrobial agent, the antibiotic gentamicin sulphate can be incorporated in hydrogel. The aminoglycoside gentamicin has been the most widely used antibiotic as it exhibits broad spectrum antimicrobial activity, excellent solubility and stability at elevated temperatures [12]. Gentamicin has been extensively used in the superficial infections of skin as it shows high effectiveness against aerobic gram-negative and some aerobic gram-positive bacteria [11]. Besides antibacterial activity, hemostasis is an important factor for rapid wound healing because, if not managed properly, the hemorrhage from the wounded area can increase the need for blood transfusion, hypovolemic shock and hypothermic coagulopathy [13]. Using a proper semi-IPN system can improve the hemostasis process, which is the first stage of wound healing. As the polymers are independent of each other while being physically interlocked to form semi-IPN, the linear polymer chains can be eluted from the network [14]. If one of the linear polymer chains in the semi-IPN has hemostatic property, e.g., collagen, gelatin, chitosan, starch, oxidized cellulose, alginate and PEG, etc. [15], it enhances hemostasis when the semi-IPN network breaks down at the site of wound. Thus, a semi-IPN hydrogel containing appropriate polymers can have huge impact on the rapid wound healing process.

In the present work, a semi-IPN CNC/PEG/PDMAA hydrogel is synthesized where PDMAA is cross-linked with N,N′-methylenebisacrylamide (NMBA), and the polymerization is supported by an initiator potassium per sulphate (KPS). The prepared hydrogel is loaded with gentamicin sulphate to observe the wound healing activity in a mice model. The significance of this research is to support wound healing by eliminating bacterial infections while releasing gentamicin in a controlled manner which can indicate better hemostatic property for faster wound healing. This hydrogel will deliver drugs to the target pathological cells to increase the effectiveness and reduce undesirable side effects.

## 2. Experimental

### 2.1. Materials

The jute fiber was collected from the local market of Tangail district, Bangladesh. The monomer Dimethyl acrylamide (DMAA), cross-linker N,N′-Methylene bisacrylamide and initiator Potassium per Sulphate (KPS) were obtained from Sigma Aldrich, Germany. Polyethylene glycol (PEG) with a molar mass of 6000 and sulphuric acid (H_2_SO_4_) were supplied by Merck, Jabalpur, Madhya Pradesh, India. The other chemicals such as ninhydrin, sodium hydroxide (NaOH), sodium chlorite (NaClO_2_), dipotassium hydrogen phosphate (K_2_HPO_4_) and potassium dihydrogen phosphate (KH_2_PO_4_) were purchased from the supplier of Loba Chemicals, Mumbai, India. The water soluble antibiotic gentamicin sulphate was obtained from Square Pharmaceuticals Limited, Dhaka, Bangladesh. The highest quality available for the chemicals was ensured, and they were used without further purification.

### 2.2. Preparation of Cellulose Nanocrystal (CNC) from Jute Fiber

The preparation of jute fiber from cellulose nanocrystal was followed as per our previous report [16]. At first, the jute fibers were washed to remove impurities. The fibers were then cut into a small size (about 2 cm) by using scissors and then milled into a fine size by using a mechanical milling machine. These fibers (25 g) were dispersed in distilled water (500 mL) for 10 min at room temperature and stirred for 2 h at 50 °C using a glass rod. There might be some extractives present in water and filtration was conducted to remove those extracts. The fibers were dried later. The dried fiber was then mercerized with 2% NaOH solution at 80 °C for 6 h with mechanical stirring followed by thorough washing. After continuous washing, when the fibers were neutralized completely, drying of those fibers was completed. After that, the dried fibers were bleached with 2 wt% NaClO_2_ at 80 °C for 4 h with mechanical stirring, washed and dried in an oven. 

The bleached fibers were additionally treated in a concentrated sulfuric acid solution (45 wt% sulfuric acid in water) at 45 °C for 10 h with mechanical stirring. The ratio of fibers to acid solution was 1:15 gL^−1^. After the treatment, the hydrolyzed cellulose samples were neutralized by 30 wt% NaOH solution in water and washed multiple times. The crystals were separated and washed by centrifugation at 8000 rpm for 10 min until the solution was completely neutralized. Finally, the cellulose nanocrystal was obtained after freeze drying for 48 h.

### 2.3. Dissolution of CNC in PEG/NaOH Solvent System

CNC powder was mixed into 20 mL of aqueous solution, containing PEG (2% *w*/*v*) and NaOH as a solvent system. The dispersion formed was allowed to be put in a deep freezer (Haier, Camden, SC, USA) at −10 °C for a period of 24 h. The next day, the obtained solid frozen mass was kept at room temperature under vigorous stirring for a period of 3 h until the solution became completely transparent. This resulting transparent solution was used to prepare the semi-IPN hydrogels.

### 2.4. Synthesis of CNC/PEG/PDMAA Semi-IPN Hydrogel

**Figure 1:**
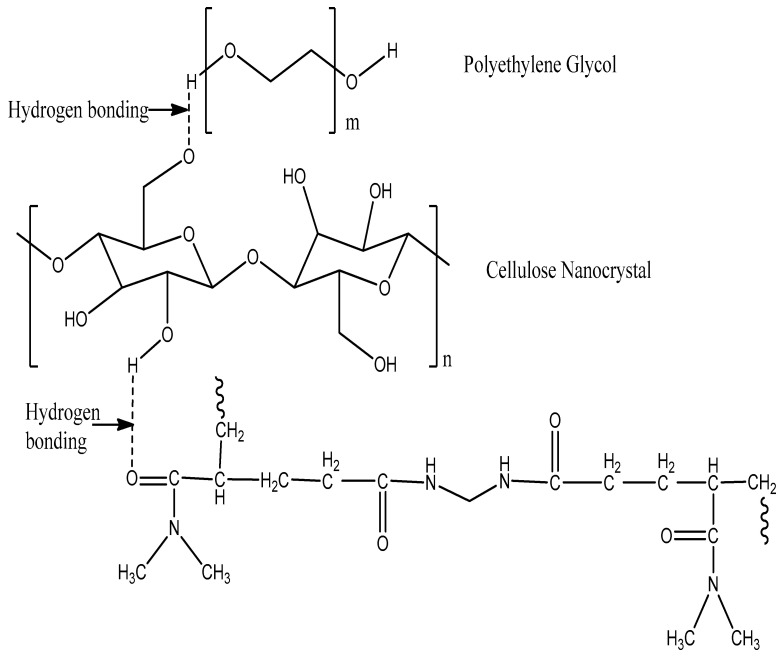
Proposed structure of semi-IPN hydrogel.

The dispersed CNC-PEG was then used to prepare the ternary hydrogel system. Different compositions of CNC 0.3, 0.8 and 1% (*w*/*v*) were used to prepare the hydrogel for comparison and the best result of formation of hydrogel was seen in the solution containing 1% *w*/*v* CNC [1]. Dimethyl acrylamide monomer (2 mL) was added to the solution followed by the addition of cross-linker NMBA (0.05 g) under constant stirring. The solution was placed in an ice bath during the addition of KPS (0.1 g) as an initiator. The mixture was stirred vigorously to ensure complete dissolution of all components. Then, the solution was transferred to a petri dish and kept in an oven at 60 °C for 2 h. After the polymerization was completed, the resulting hydrogels were taken out of the oven in film form and freeze dried (Supporting Information, Figure S1). The gel was then used for a further characterization process. The possible structure of the hydrogel is shown in Figure 1, and the cross-linking of monomer with cross linker NMBA [2] is given in Supporting Information (Figure S2).

### 2.5. Characterization Techniques

Attenuated Total Reflectance (ATR) spectra of the samples were obtained using the IR spectrophotometer (IR prestige-21, Shimadzu Corporation, Kyoto, Japan) in the range of 4000–400 cm^−1^. The particle size of CNC was observed by Transmission Electron Microscope (TEM) (JEM-1400 Plus, Tokyo, Japan). Thermal properties of the samples were carried out by thermo gravimetric analysis (TGA) (TGA-50, Shimadzu, Tokyo, Japan) under nitrogen atmosphere in aluminum cell with a temperature rate of 10 °C/min up to 600 °C from room temperature. X-ray powder diffraction (XRD) analysis was investigated in a Rigaku Ultima IV X-ray diffractometer (Rigaku Americas Corporation, Tokyo, Japan) using Cu-Kα radiation with a scan speed of 5°/min ranging from 20° to 80°.

To observe the surface morphology of hydrogel and CNC, The Carry Scope JCM-5700 Scanning Electron Microscope (JEOL, Tokyo, Japan) was used and, for observing the morphology of the drug loaded hydrogel, JEOL JSM-6490LA, an analytical scanning electron microscope was used. The sputtering machine used for this machine was a JEOL JFC 1600 auto fine coater.

The UV-1700 Pharmaspec (Shimadzu, Kyoto, Japan) was used for the determination of drug concentration in solution. For gentamicin drug, a wavelength of 400 nm was used.

### 2.6. Swelling Test of CNC/PEG/PDMAA Semi-IPN Hydrogel

The swelling behavior of the hydrogel was determined at first by completely drying the hydrogel and by immersion of the hydrogel both in distilled water at room temperature and in pH 7.4 at 37 °C, to compare the swelling property in both of the cases. About 20 mg of the sample (1.5 cm × 1.5 cm) was weighed and immersed in around 20 mL of solution for observation of swelling property. The gel was gently wiped and weighed at various time intervals for the test.

The swelling studies were carried out until equilibrium in swelling was reached. The swelling percentage was calculated using the following equation:

S% = ((M_t_ − M_0_)/M_0_) × 100% (1)

Here, M_t_ and M_0_ refer to the weight of the swollen hydrogel at time t and initial time, respectively.

The equilibrium percent swelling S_eq_ after the hydrogel had swollen to equilibrium in the swelling media was calculated using the following formula:

S_eq_% = ((M_eq_ − M_0_)/M_0_) × 100% (2)

where M_eq_ is the mass of the swollen hydrogel sample at equilibrium.

The water absorbed by CNC/PEG/PDMAA Semi-IPN hydrogel is quantitatively represented by equilibrium water content (EWC) [3,4], where

EWC = (M_eq_ − M_0_)/M_eq_ (3)

### 2.7. Gentamicin–Ninhydrin Assay

Gentamicin poorly absorbs ultraviolet and visible light, and so the indirect spectrophotometric method was used for assaying the gentamicin sulphate standard curve and loading the drug onto the gel. Here, ninhydrin was used to form a complex with gentamicin which is basically based on a ninhydrin reaction with primary and secondary amines present in the gentamicin, producing a colored solution [5]. In this method, a clear spectrum was shown whereas, in the case of using only gentamicin, no significant spectrum was observed.

The standard curve was prepared using this gentamicin–ninhydrin complex, where seven different concentrations of gentamicin (20, 40, 80, 100, 200, 400 and 500 mg/L) were used. The phosphate buffer solution (PBS) was prepared in the lab following the standard of the European Pharmacopoeia and mixed with ninhydrin by heating, to form ninhydrin PBS reagent. In each case, 5 mL of antibiotic solution was mixed with 1.5 mL of ninhydrin PBS reagent by vortexing (30 s) and then heated at 95 °C for 15 min in a water bath [6]. The solutions were then cooled in an ice water bath and the required amount of the solutions was subjected to UV reading at 400 nm against the ninhydrin PBS reagent as a background reading, and thus the standard calibration curve was prepared. The loading and release profile of the drug was quantified in UV visible spectrophotometer using the same method of forming the ninhydrin complex with the antibiotic, and then the efficiencies were calculated.

### 2.8. Loading of Gentamicin Sulphate on CNC/PEG/PDMAA Semi-IPN Hydrogel Film

The loading of the antibiotic gentamicin sulphate was performed by soaking the hydrogel film in the gentamicin solution. In this experiment, 10 mg of prepared hydrogel film were soaked in gentamicin sulphate solution of varying concentrations (20, 40, 100, 200 and 300 mg/L). The solutions were placed in a reciprocating shaker at 90 rpm for 24 h [7] for facilitating the drug uptake. The absorbance values of unloaded drug measured by UV Visible Spectrophotometer at 400 nm, examined by ninhydrin assay, helped to determine the efficiency of the drug. The percentage of loading efficiency was calculated using the following formula [8]:

(4) $$\mathrm{Loading}\;\mathrm{Efficiency}\;\%=\frac{\mathrm{Initial}\;\mathrm{Drug}\;\mathrm{Concentration}-\mathrm{Drug}\;\mathrm{concentration}\;\mathrm{in}\;\mathrm{supernatant}}{\mathrm{Initial}\;\mathrm{Drug}\;\mathrm{Concentration}}\times 100\%$$

### 2.9. In Vitro Cytotoxicity and Biocompatibility Study by Cell Culture

A cytotoxicity test was designed to determine the toxicity of a compound to cells, either qualitatively or quantitatively. For the prepared hydrogel, two cell lines were used for testing cytotoxicity. They were BHK-21, a baby hamster kidney fibroblast cell line, and Vero cell line, kidney epithelial cells extracted from an African green monkey.

In brief, BHK-21 cells and Vero cells were maintained separately in DMEM (Dulbecco’s Modified Eagles medium) containing 1% penicillin–streptomycin (1:1) and 0.2% gentamycin and 10% fetal bovine serum (FBS). Both cells (3 × 10^4^/200 µL) were seeded onto 48-well plate and incubated at 37 °C + 5% in a CO_2_ (CO_2_ Incubator, Nuaire, Plymouth, MN, USA) environment for 24 h. The next day, 50 µL of sample (autoclave) was added to each well. Cytotoxicity was examined under an inverted light microscope (trinocular microscope with camera, Optika, Ponteranica, Italy) after 48 h of incubation. Duplicate cells were used for each sample.

### 2.10. Antimicrobial Activity and In Vitro Release Profile

The analysis of antibacterial activity was studied using the agar diffusion method by Kirby-Bauer, 1985 [7]. In this method, generally, the test agar plate was wiped by a standardized concentration of test where paper disks with different concentration samples were placed on the lawn of bacteria. After overnight incubation, the zone of inhibition was identified and the diameter of the zone around the disk was measured.

The antibacterial activity was examined for both gram-positive *Staphylococcus aureus* and gram-negative *Escherichia coli* bacteria. A hole was made in the Agar broth to keep the drug loaded sample, and the bacterial colonies were swabbed in the agar broth and, after that, left for 24 h for incubation. The inhibition zones were checked after 24 h. The measurement of the inhibition zones was made with a ruler under the surface of the plate without opening the lid.

For determining the in vitro release profile, the optimum drug loaded hydrogel (1.5 cm × 1.5 cm) was placed in a phosphate buffer solution of pH 5.5 and pH 6.0 in a conical flask. The buffer solutions of pH 5.5 and 6.0 were prepared in the lab using the European Pharmacopoeia guideline. The flask was placed in a shaker which was maintained at 37 °C and 100 rpm. After a predetermined time interval, 3 mL of the aliquots were withdrawn from the solution, which was replaced by the same amount of freshly prepared buffer solution. The aliquots represent the released amount of gentamicin in the solution, which was quantified using ninhydrin assay by UV Visible spectrophotometer at 400 nm wavelength range.

### 2.11. In Vivo Wound Healing Evaluation

Experiments of in vivo wound healing evaluation were conducted in two groups: a normal surgical gauge (control group) and a drug loaded hydrogel in mice model where each mouse weighed around 20–25 gm. The mice were maintained under standard pellet and water conditions along with controlled environmental conditions. The mice were anesthetized by Ketamin HCl injection (10 mg/kg), injected into the lower abdomen to make them unconscious for about 15 min. An electric trimmer was used to shave the dorsal fur 5 mm away from the ears of the mice and a wound approximately 1 cm × 1 cm was cut [17] in each mouse. For creating a full thickness wound with minimal bleeding, both the epidermal and dermal layers were removed. The wound areas were sterilized using 70% ethanol.

Before placing the gel onto the wounded area, the gel (1.5 cm × 1.5 cm) was immersed in saline solution for a certain period of time. The efficacy of the wound healing was determined by observing wound morphology, re-epithelialization and wound contraction with naked eye observation [9]. The evaluation of the healing process was examined on the fourth, eighth and twelfth day, consecutively, and photographs were taken for examining the size of the wound. The relative wound size reduction was calculated using the following equation [10]:

(5) $$\mathrm{Relative}\;\mathrm{wound}\;\mathrm{size}\;\mathrm{reduction}\;(\%)=\frac{A_{0}-A_{t}}{A_{0}}\times 100$$

Here, *A_o_* is wound size at initial time, and *A_t_* is the wound size at any time ‘*t*’.

## 3. Results and Discussion

### 3.1. ATR Spectra Analysis

**Figure 2:**
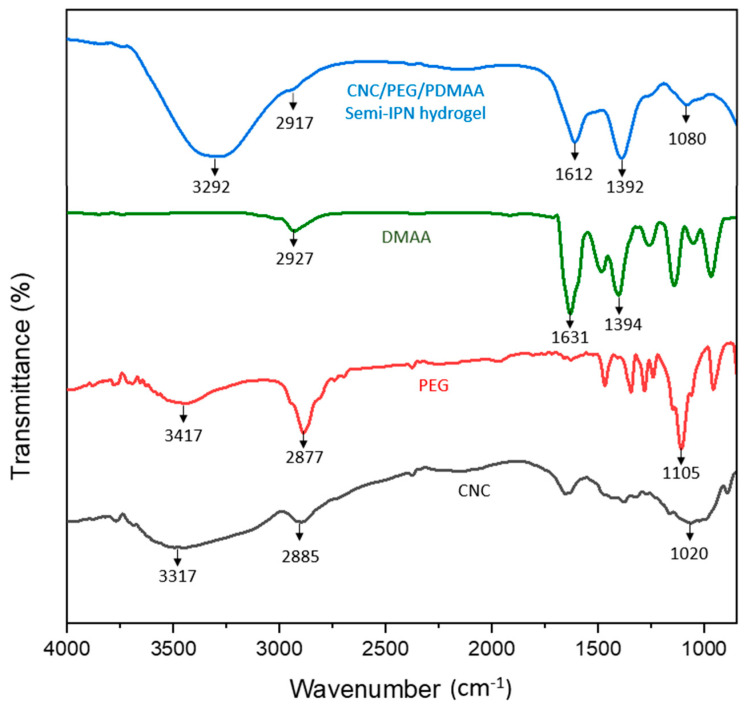
ATR Spectra of CNC/PEG/PDMAA semi-IPN hydrogel, DMAA, PEG, CNC.

The ATR spectra of CNC, PEG, DMAA and the semi-IPN hydrogel are shown in Figure 2.

The major characteristic peaks for cellulose were at 3317 cm^−1^, due to –OH stretching, 2885 cm^−1^, which corresponded to C-H stretching in sp^3^ hybridized bond, 1420 cm^−1^ due to symmetric bending in –CH_2_ and 1020 cm^−1^, which corresponded to C-O-C asymmetric vibration that was associated with cellulose. The major characteristic peaks for PEG were at 3417 cm^−1^ due to –OH stretching, 2877 cm^−1^ for stretching vibration of the –CH group, 1465 cm^−1^, which corresponded to the deformation vibration of the C-H group, 1385 cm^−1^ due to –OH bending vibration and 1105 cm^−1^, which was due to C-O stretching. Moreover, the major characteristic peaks for DMAA were at 2927 cm^−1^ due to C-H stretching in sp^3^ hybridized bond, 1631 cm^−1^, which corresponded to C=O stretching vibration of amide, 1394 cm^−1^, due to the C-N stretching vibration of amide and a medium strong peak at 1500 cm^−1^ for the C=C group [11].

The ATR spectrum of the semi-IPN hydrogel contained a major characteristic peak of CNC, PEG and DMAA. Some shifting of the peaks had occurred, which might prove H-bonding and complete polymerization of the monomer. There was a broad absorption peak at 3292 cm^−1^ due to hydrogen bonding in the –OH group because the peak was slightly shifted and broadened from the raw material CNC and PEG, and hence this confirmed the hydrogen bonding between CNC and PEG in the semi-IPN hydrogel. There was a slight absorption peak at 2917 cm^−1^ region due to C-H stretching in sp^3^ hybridized bond. The C=O stretching vibration of amide shifted from 1631 cm^−1^ to 1612 cm^−1^ after the formation of hydrogel, which suggested the existence of an inter- or intra-molecular interaction of DMAA with other raw materials. The C-N stretching of the amide group showed that the absorbance peak at 1392 cm^−1^ and absorbance peak at 1080 cm^−1^ was due to the C-O-C asymmetric vibration, which might be associated with cellulose. In the hydrogel, the peak of the C-H group at 1465 cm^−1^, shown in the PEG spectrum, disappears completely because this peak has merged with the nearest C-N stretching peak (1392 cm^−1^) of the amide group. In DMAA, there was a medium weak peak at 1500 cm^−1^ for the C=C group which disappeared in the hydrogel, confirming the complete polymerization of the monomer.

### 3.2. X-ray Diffraction Pattern Analysis

**Figure 3:**
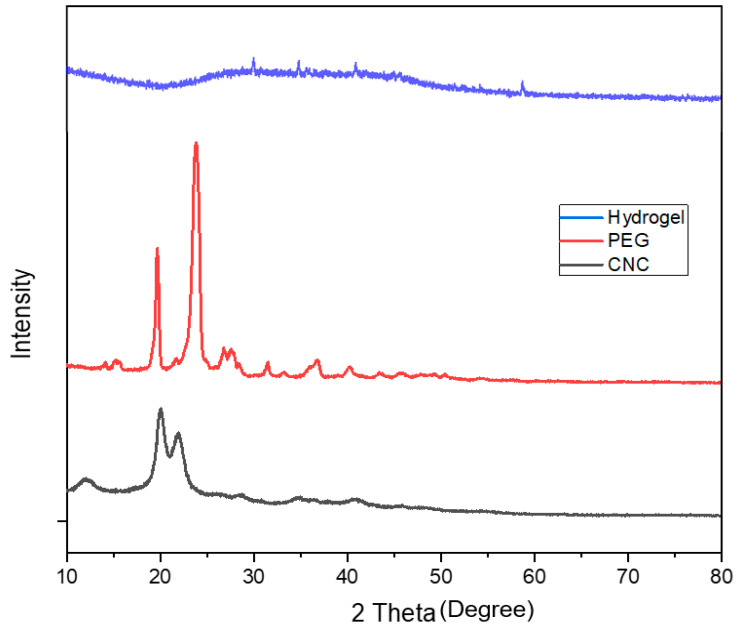
XRD pattern of CNC/PEG/PDMAA semi-IPN Hydrogel, PEG and CNC.

Figure 3 shows the XRD pattern of CNC, PEG and CNC/PEG/PDMAA semi-IPN hydrogel.

The characteristic crystalline peak for CNC centered at 2 theta = 20.5°; 23° was observed in the diffractogram of CNC [12]. Cellulose shows orderly hydrogen bonding arrangement among the molecules, and hence during acid hydrolysis, acid could not penetrate and hydrolyze the crystalline region. Therefore, it cleaved only the amorphous region, leaving the crystalline area intact, which resulted in high degree of crystallinity of CNC [18,19]. PEG was observed to show major XRD peaks at 2 theta = 19.39°, 23.56° and a few minor peaks at 2 theta = 27.2°, 36.4°, 39° and 45.2°, respectively, which was similar to the data in the previous literature [20].

The X-ray diffraction pattern in the semi-IPN hydrogel showed no characteristic major peak of CNC or PEG due to the small amount of the constituent polymers. No peaks of CNC were observed in the hydrogel and some minor peaks of PEG were shown. After forming semi-IPN, a drastic change in the typical characteristic peak of the crystalline CNC was seen, which indicated that the crystal structure of CNC was changed after the formation of semi-IPN and confirmed the uniform dispersion of the constituent polymers [21].

### 3.3. Thermogravimetric Analysis

**Figure 4:**
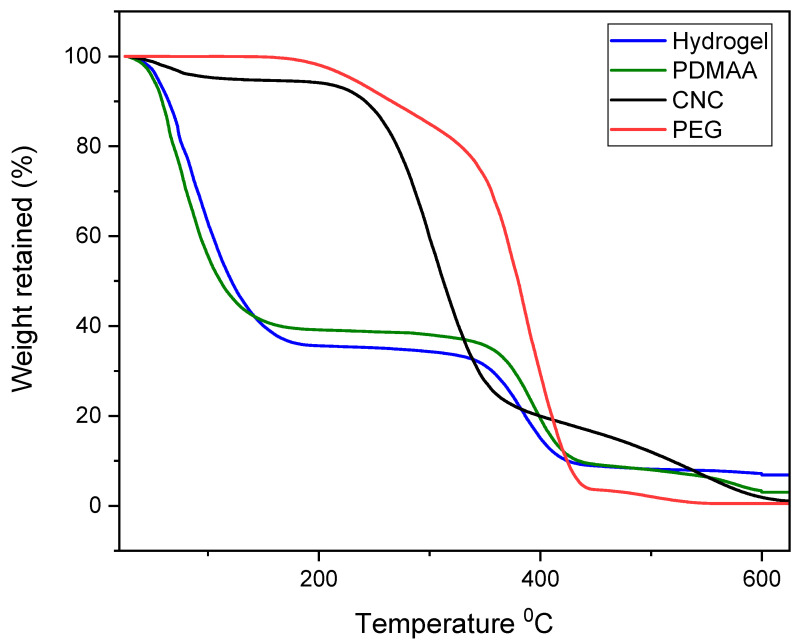
TGA curves of CNC, PEG, PDMAA and CNC/PEG/PDMAA semi-IPN hydrogel.

The thermograms of the samples CNC, PEG, PDMAA and CNC/PEG/PDMAA semi-IPN hydrogel are shown in Figure 4.

In the thermogram of CNC, first thermal degradation or weight loss of nearly 5% is in the range of 25 °C to 150 °C was seen. This degradation might be due to the moisture evaporation of the CNC. Afterwards, a sharp thermal degradation could be seen in the temperature range of 210 °C to 400 °C due to the pyrolysis of the cellulose. In this phase, volatile hydrocarbon might be liberated from the cellulose. Here, the weight loss was nearly 65%. Finally, frequent weight loss was observed. This could be attributed to the fact of the decomposition of the remaining heavy component in cellulose, mainly from lignin [22]. Here, almost all weight of CNC was lost. PEG had a single thermal degradation stage. It started to degrade at around 320 °C and ended at 420 °C. In this degradation, almost 95% of the weight loss of PEG was observed, and with higher temperature, complete weight loss was observed.

The TGA curve of the ternary hydrogel system showed multistage degradation due to the presence of different polymers in the system. The first stage degradation was seen approximately at 150 °C, which might be due to the moisture evaporation of the polymeric constituents. After that, the second stage degradation was up to 350 °C and, here, around 67% weight loss was observed. Successive cleaving of the backbone of the polymeric hydrogel could be the reason for this degradation stage [23].

The third stage degradation occurred from 350 °C to 420 °C, which was due to the presence of a small amount of PEG. From the TGA curve of pure PEG it could be seen that the degradation started from 320 °C. However, in this hydrogel, it started to degrade at 350 °C, which might ensure the improvement of the thermal stability of PEG. The weight loss remained nearly constant when the temperatures were higher than 420 °C. From the thermogram of PDMAA, it can be clearly seen that the thermal stability of the hydrogel is mainly governed by the PDMAA, as this polymer is highest in composition compared to other polymers present in hydrogel. If we could compare the overall stability of the hydrogel and the constituent polymers individually, then it was seen that overall weight loss is approximately 90% at 600 °C for the hydrogel sample, whereas the polymer constituents exhibited almost 100% weight loss at this temperature.

### 3.4. Morphology and Particle Size Analysis

**Figure 5:**
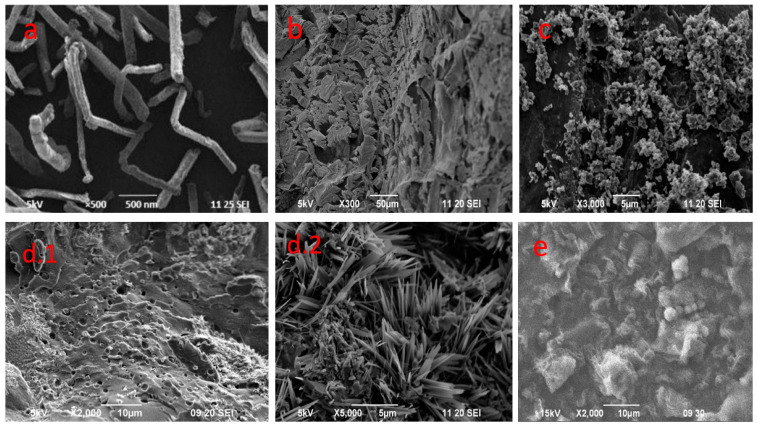
SEM image of (**a**) CNC, (**b**) hydrogel with 0.3% CNC, (**c**) hydrogel with 0.8% CNC, (**d1**) hydrogel with 1% CNC 2000×, (**d2**) hydrogel with 1% CNC 5000× and (**e**) gentamicin loaded hydrogel with 1% CNC.

The surface morphology of the samples was identified by SEM analysis, shown in Figure 5. The SEM image of CNC (Figure 5a) gave a clear external appearance of the cellulose nanocrystal by showing the Nano-rod-like structure. The hydrogel was prepared first with three different compositions of CNC (0.3, 0.8 and 1%), which are shown in Figure 5 b, c, d1, d2). The SEM images indicated that the hydrogel prepared with 1% CNC had three-dimensional porous structure. The pores and spongy surface in the hydrogel helped to increase the swelling property of the product. The pores could be the region of water permeation and interaction site of the water molecules with hydrophilic groups of the hydrogel. Thus, this composition was used for further analysis as it could be easier to load drugs in this composition. The SEM image of hydrogel after gentamicin drug loading at its optimum loading efficiency is shown in Figure 5e. This image clearly shows the entrapment filling the pores, compared to the SEM image of the hydrogel in Figure 5d.1. The comparison clearly shows that the structure of the gel was not affected by the incorporation of the drug onto the hydrogel [10].

**Figure 6:**
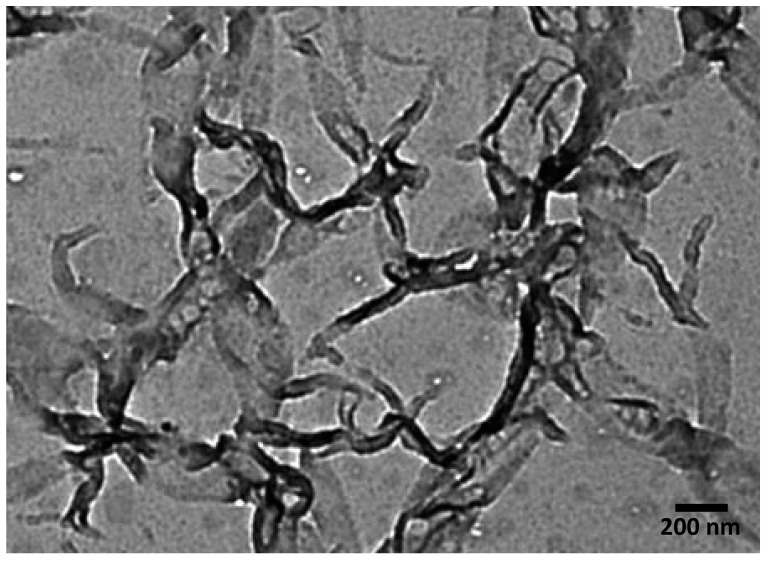
TEM image of CNC.

The particle size for CNC was determined by TEM analysis, which is shown in Figure 6. The particle size of the nanocrystal was found to be 40–80 nm.

### 3.5. Swelling Study

**Figure 7:**
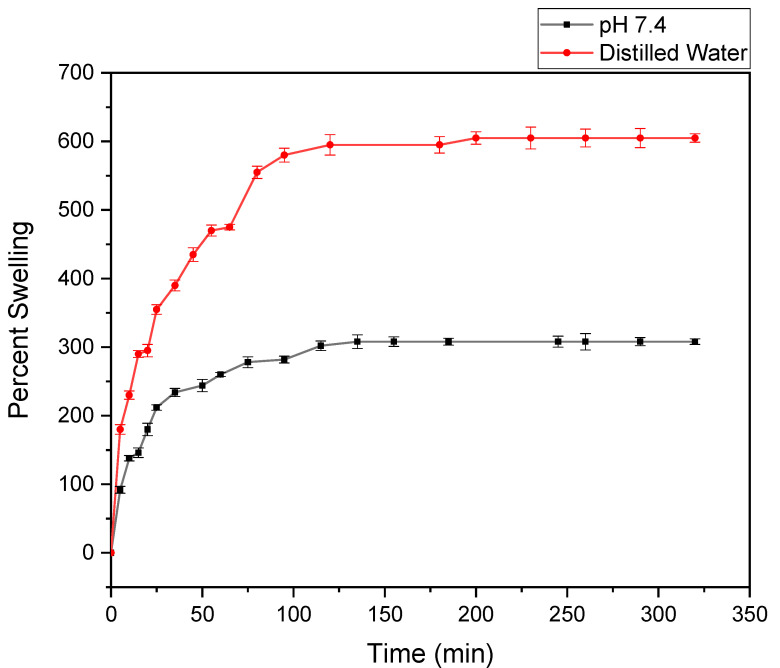
Percent swelling vs. time curve of CNC/PEG/PDMAA semi-IPN hydrogel in water and pH 7.4 phosphate buffer.

The swelling behavior of the hydrogel had been studied as a function of time and is shown in Figure 7. The figure shows that the percent of swelling in distilled water was 605%, and in phosphate buffer it was 308%, and the equilibrium was reached within 4–5 h. The hydrophilic ability of the functional groups and effective crosslink density of the hydrogel mainly governed the swelling property of any hydrogel. Cellulose acted as a multifunctional cross-linker to form more junctions in hydrogel, followed by an increase in crosslinking density in hydrogel. This could result in the decrease in swelling capacity. The increased number of crosslinks in the polymer segments could reduce free space available for accommodation of the incoming solvent. This was because of the rigidity of the chain that resulted by the crosslinking and, hence, restricted their relaxation [24]. In this system, all the components were hydrophilic and, for this reason, the water absorption behavior of the hydrogel was governed by the concentration of the components in the feed mixture.

The value of equilibrium water content (EWC) was found in the range of 0.8, which showed that the synthesized hydrogels could be used as biomaterials on any biomedical application, because EWC was bigger than the percent of water content value of the body, about 0.6 [25].

### 3.6. Cytotoxic Effect Analysis

**Figure 8:**
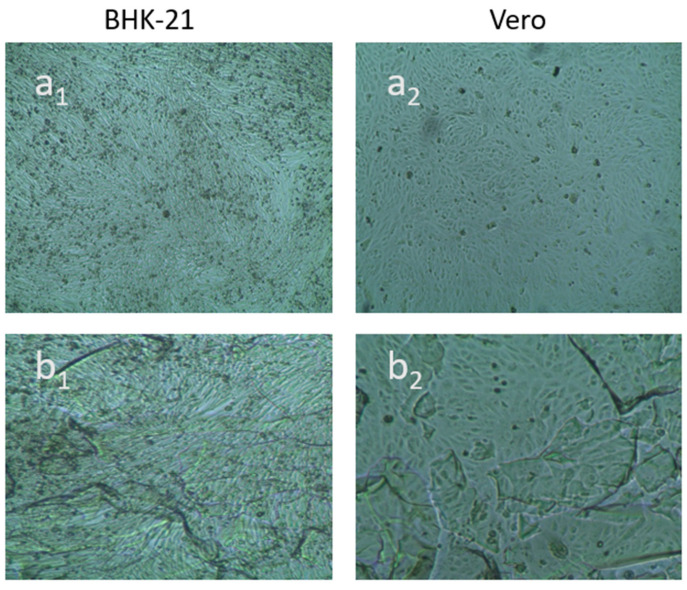
Optical microscopic images of BHK-21 and Vero cell lines treated with semi-IPN hydrogel; (**a1**) control and (**b1**) hydrogel after 48 h for BHK-21 cell, (**a2**) control and (**b2**) hydrogel after 48 h for Vero cell.

Cytotoxicity of the hydrogel was examined under an inverted light microscope after 48 h of incubation to both the BHK-21 and Vero cell line. Cytotoxicity of the control was also measured for each cell. The two cell lines were observed for 48 h to examine any possible morphological changes, areas of cellular lysis and cell death [26]. Table 1 shows the results of the cytotoxicity tests conducted using the BHK-21 cell line and Vero cell line. No cytotoxicity was observed for the hydrogel sample after 48 h observation on both the BHK-21 cell line and Vero cell lines, as the percentage of survival for both the cells was greater than 95% (Figure 8). The prepared gel survived the cell line and, therefore, the hydrogel could be applied to animal cell wound healing.

### 3.7. Gentamicin Drug Loading

#### 3.7.1. Standard Curve of Gentamicin Sulphate

**Figure 9:**
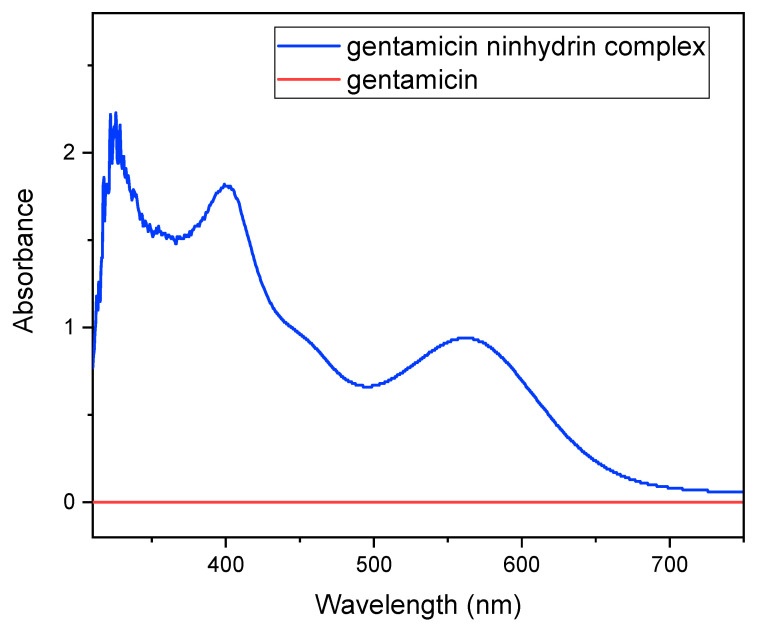
Spectrophotometric curve of gentamicin and the gentamicin–ninhydrin complex.

The standard curve of the gentamicin sulphate was prepared by forming gentamicin ninhydrin complex at first because gentamicin alone does not show a significant spectrum in the UV-Visible spectrophotometer. Five mL of gentamicin sulphate solution (400 mg/L) were mixed with 1.5 mL of ninhydrin reagent (2 mg/mL) to form a complex which shows nearly three maxima in the spectrum (Figure 9). Figure 9 shows three maxima near 320, 400 and 550 nm in the gentamicin–ninhydrin complex where, as in the gentamicin solution, no spectrum was observed. The absorbance values at 400 nm of the complex mixtures remained virtually unchanged for at least 4 h, and hence this wavelength was taken as standard [5]. Then, at the 400 nm range, the standard calibration curve was drawn.

**Figure 10:**
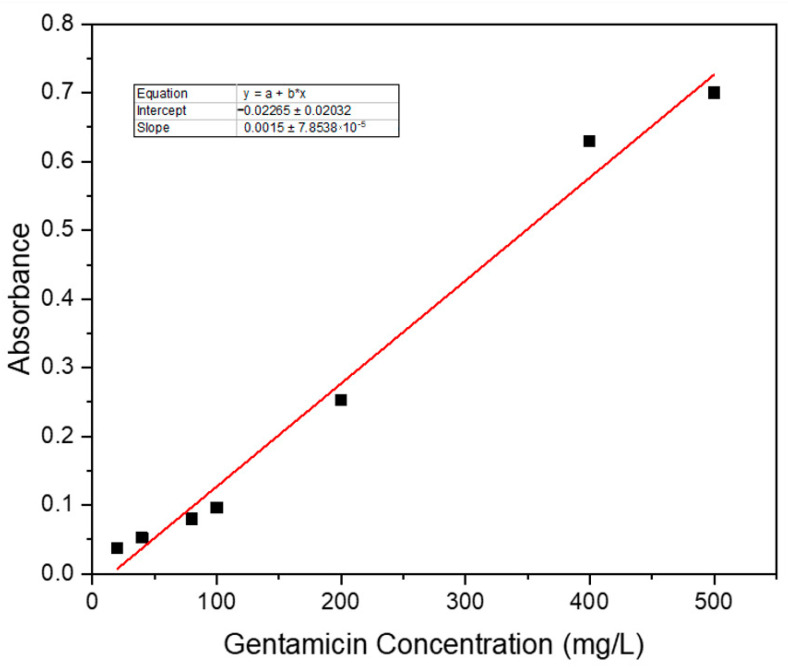
Standard calibration curve for gentamicin sulphate.

Seven different known concentrations of gentamicin sulphate (20, 40, 80, 100, 200, 400 and 500 mg/L) were prepared by forming the ninhydrin complex and were subjected to 400 nm to obtain the absorbance values, and thus was the standard curve obtained (Figure 10) (R^2^ value = 0.9899), which was further used for quantifying the drug loading and release profile.

#### 3.7.2. Drug Loading Performance and Optimum Drug Loading Efficiency

The suitable loading of gentamicin sulphate could be quantified by loading different concentrations of drugs onto the hydrogel film. The images of hydrogels before and after drug loading are presented in the Supporting Information (Figure S1). The hydrogel film was soaked in a gentamicin sulphate solution of varying concentrations (20, 40, 100, 200 and 300 mg/L). Then, the absorbance values of unloaded drugs in the supernatant solution helped to quantify the loading efficiency of the drug. These absorbance values were measured with a UV-Visible spectrophotometer at 400 nm compared to the standard calibration curve of gentamicin sulphate. The loading efficiencies of 20, 40, 100, 200 and 300 mg/L gentamicin sulphate were 87.17, 62.5, 61.16, 40.15 and 32.19%, respectively. Table 2 shows the loading performance of gentamicin sulphate for varying concentrations.

**Figure 11:**
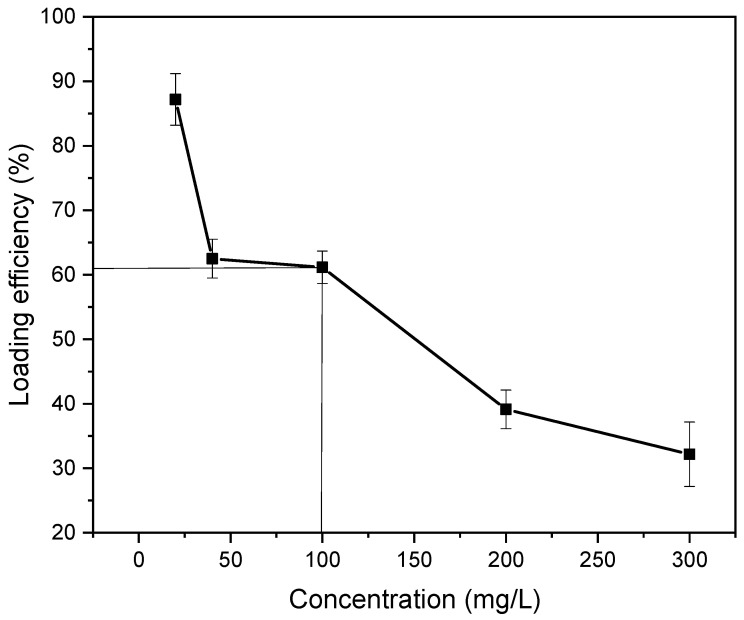
Optimum drug loading efficiency determination.

The optimum loading efficiency was identified as 61.16% with a gentamicin sulphate concentration of 100 mg/L (Figure 11). The optimum loading efficiency was determined to be 61.16% because, in this percentage, the maximum amount of drug was loaded with the highest efficiency. The crosslinking of this semi-IPN hydrogel film resulted in some void spaces for the physical entrapment of the drug in these spaces [27]. After 100 mg/L concentration, a sharp decrease in drug loading efficiency was seen, and hence it could be concluded that the highest drug entrapment inside the void spaces occurred in this efficiency.

### 3.8. Antimicrobial Activity Study

**Figure 12:**
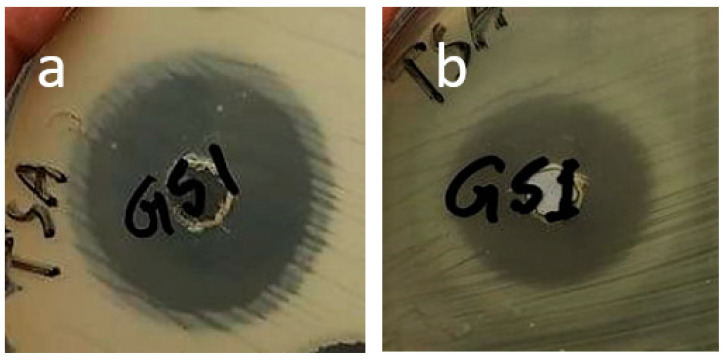
Antibacterial activity of drug loaded hydrogel film showing the inhibition zone for (**a**) *S. aureus* and (**b**) *E. coli* bacteria.

Antibacterial study was observed in one gram-negative (*E. coli*) and one gram-positive (*S. aureus*) bacteria. For assessing the activity pattern, strains of the bacteria were swabbed in the agar broth and left for incubation for 24 h, resulting in an inhibition zone in both cases (Figure 12), which was measured using a millimeter scale. The zone of inhibition observation showed antibacterial activity for the gentamicin loaded hydrogel film, and hence it could be concluded that the hydrogel film could be used for wound healing purposes, which could inhibit the bacterial growth in the wound exposure. The zone of inhibition for both the bacteria is listed in Table 3.

### 3.9. In Vitro Release Profile

Slightly acidic solutions were used for determining the release profile because, during the healing of acute wounds, generally, a temporary physiological acidosis is seen due to the generation of different organic acids and increased demand for oxygen during the healing process combined with a stasis of tissue perfusion, increasing the local carbon dioxide in wounds [28]. Therefore, at acidic pH 5.5 and 6.0, a better release profile could be understood.

**Figure 13:**
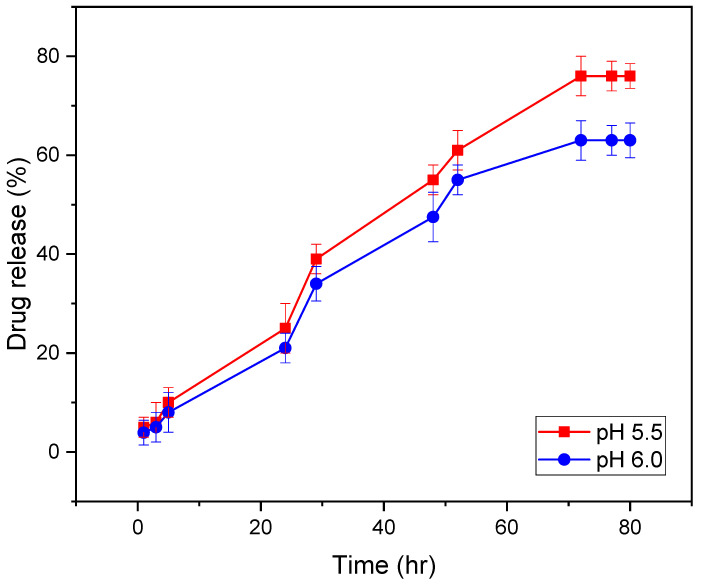
In vitro drug release profile of gentamicin sulphate from hydrogel in phosphate buffer solution at pH 5.5 and pH 6.0.

The release of the drug under in vitro conditions at various time intervals, up to an 80 h period, showed the highest percentage of release of 76% within 72 h (Figure 13) at pH 5.5 and release of 63% within 72 h at pH 6.0. Figure 13 shows drug release in a manner which indicated homogeneous dispersion of the drug in the polymer matrix, which resulted in a sustained drug release in acidic pH and confirmed that the drug could be released in the acidic environment of the wounded area.

### 3.10. In Vivo Wound Healing in Mice Model

**Figure 14:**
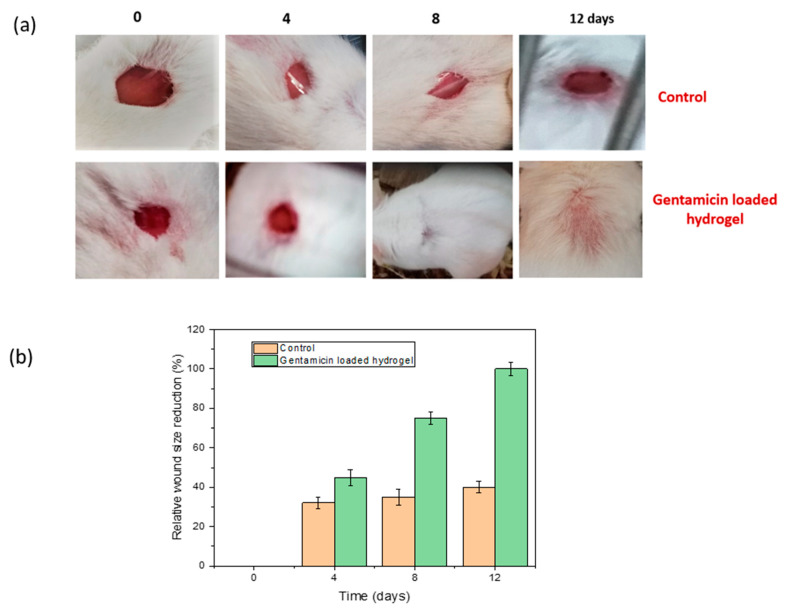
(**a**) Photographs of wounds treated with normal gauze and gentamicin loaded hydrogel. (**b**) Evaluation of relative wound size reduction.

The efficacy of the prepared hydrogel film was determined by the responsiveness of the faster wound healing which had been observed using the 12 day mice model experiment [29]. The wound closure observation continued on day 0, 4, 8 and 12, both for the control mouse which was treated without drugs and another mouse with optimum drug loaded hydrogel film. The results are shown in Figure 14a,b with a comparison of relative wound size reduction. The control mouse which was treated with normal gauze showed no significant size reduction in the wound. Within this 12-day experiment period, only 40% wound closure was observed for the control mouse, whereas the mouse treated with optimum drug loaded semi-IPN hydrogel indicated noteworthy wound closure within this time period. On the eighth day, the hydrogel loaded with drug showed a wound size reduction of 75%, which was significantly higher compared to the control. Less scabbing was observed in the case of the wound treated with drug loaded hydrogel and, on the twelfth day, complete wound closure of 100% was exhibited with complete growth of the skin tissues.

## 4. Conclusions

In this research work, we tried to develop a biocompatible hydrogel which could be in contact with bodily organs with minimum damage to the surrounding tissues and without triggering undesirable immune responses. The biocompatibility of the semi-IPN hydrogel along with the faster wound healing response, which was confirmed by the in vivo testing, and lack of toxicity have been taken advantage of in wound dressings, and hence this gentamicin loaded CNC/PEG/PDMAA semi-IPN hydrogel can be successfully used in various wound healing purpose. The complete polymerization and formation of semi-IPN was confirmed by ATR analysis. The morphological studies showed their efficacy in drug loading, with an optimum gentamicin sulphate loading efficiency of 61.16% and with a drug concentration of 100 mg/L. The antibacterial activity against *S. aureus* and *E. coli* bacteria confirms their suitability for wound healing. In vitro drug release of 76% at pH 5.5 within 72 h followed by a successful in vivo testing in a mice model, with a recovery within 12 days, give this work a possible scope for wound healing management. Overall, the easy preparation method, low operational cost and outstanding wound healing properties of this advanced hydrogel compound have made it a potential biomaterial for wound dressing.

## Figures and Tables

**Table 1:** Cytotoxic effect analysis of CNC/PEG/PDMAA semi-IPN hydrogel.

Sample ID	Survival of BHK-21 Cell	Survival of Vero Cell	Remarks
Control (−)	100%	100%	No cytotoxicity
Control (+)	>95%	>95%	No cytotoxicity
Hydrogel sample	>95%	>95%	No cytotoxicity

**Table 2:** Drug loading performance of hydrogel film in different concentrations.

Gentamicin Sulphate Concentration (mg/L)	Loaded Amount of Gentamicin Sulphate in 24 h (mg)	Loading Efficiency (%)	Standard Deviation(± %)
20	17.43	87.17	3.26
40	25	62.5	2.44
100	61.16	61.16	2.04
200	80.3	40.15	2.67
300	96.57	32.19	4.08

**Table 3:** Zone of inhibition (mm) in drug loaded hydrogel sample.

Sample	Bacteria Specific Diameter (mm) of Inhibition Zone	
	*Staphylococcus aureus*	*Escherichia coli*
Drug loaded hydrogel film	25	23

## Data Availability

Not applicable.

## Supplementary Materials

The following supporting information can be downloaded at: https://www.mdpi.com/article/10.3390/gels8060340/s1, Figure S1: Images of CNC/PEG/PDMAA semi-IPN hydrogel-before and after freeze drying; and drug loading; Figure S2: Cross-linking of DMAA to give PDMAA.
