# Supplementary material for: Advanced CNC/PEG/PDMAA Semi-IPN Hydrogel for Drug Delivery Management in Wound Healing
Source: Gels. 2022 May 30;8(6):340. doi: 10.3390/gels8060340 (PMC9222527; doi:10.3390/gels8060340)
Supplement: Supplementary file 1 [file gels-08-00340-s001.zip › gels-1726390-supplementary.pdf]

gels

Supplementary Materials

Advanced CNC/PEG/PDMAA Semi-IPN Hydrogel for Drug Delivery Management in Wound Healing

Samia Afrin [1,2], Md. Shahruzzaman [1,*], Papia Haque [1], Md. Sazedul Islam [1,3], Shafiul Hossain [1,4,5], Taslim Ur Rashid [1,6], Tanvir Ahmed [1], Makoto Takafuji [7] and Mohammed Mizanur Rahman [1]

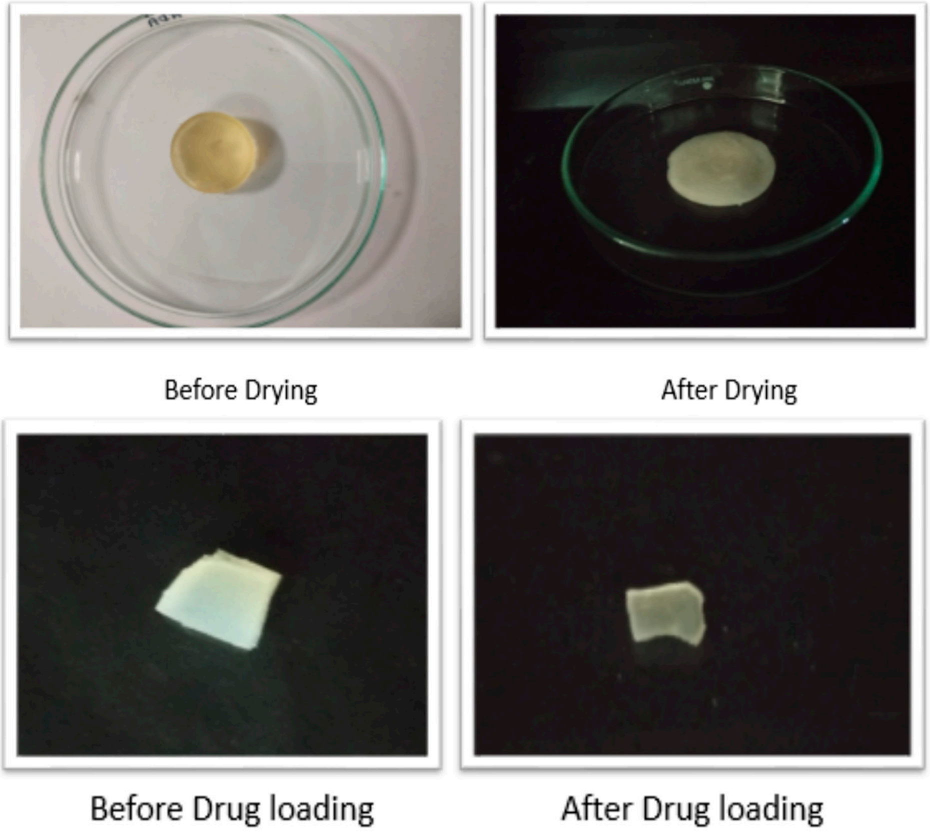

Figure S1. Images of CNC/PEG/PDMAA semi-IPN hydrogel - before and after freeze drying; and drug loading.

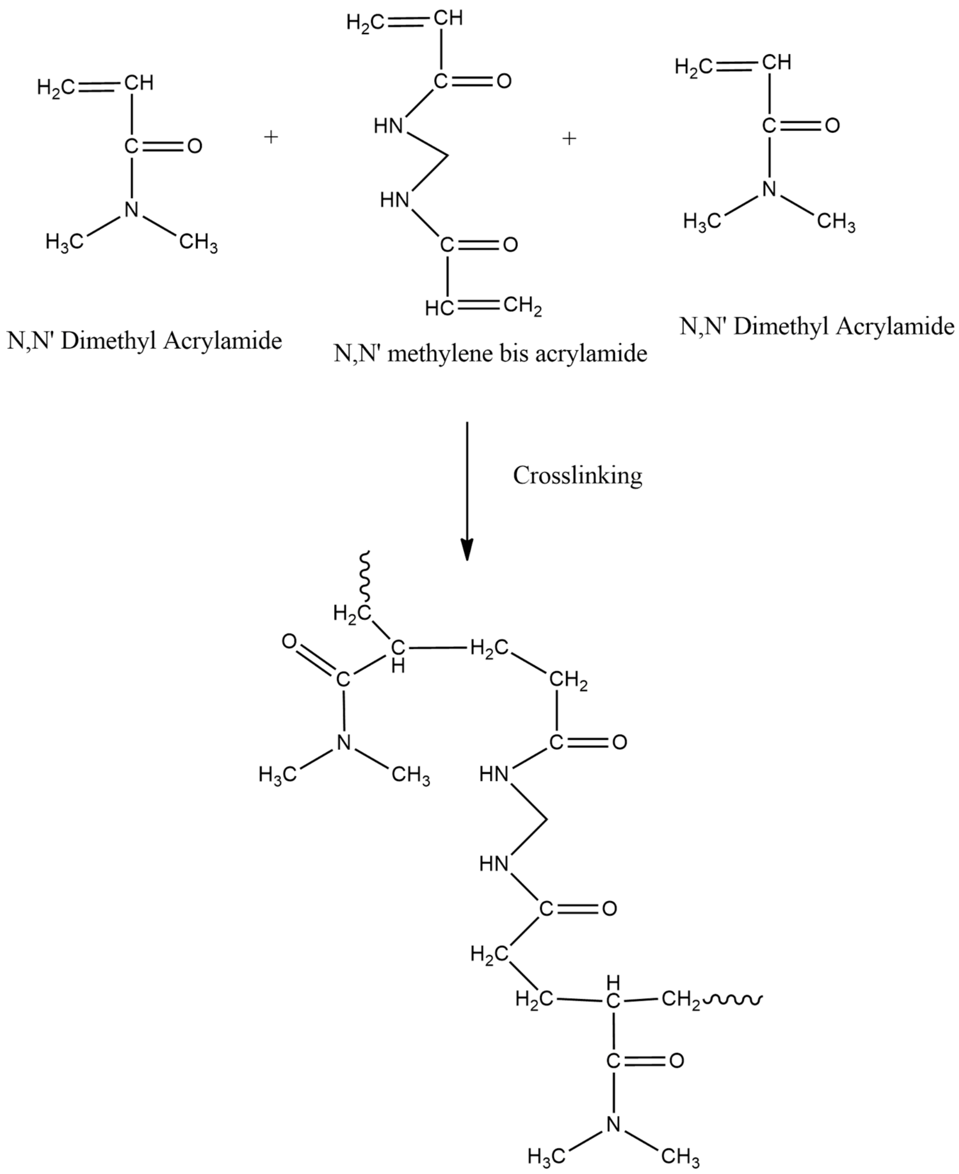

Figure S2. Cross-linking of DMAA to give PDMAA.
